# To Junior Dentists

**Published:** 1887-09

**Authors:** 


### TO JUNIOR DENTISTS.

We have received a number of letters asking that the series of letters to students and young dentists be revived. We recognize the fact that there are many of our readers who need and desire elementary information, and it is our purpose to comply with the requests made at as early a date as is practicable.

